# Les médecins prestataires à la première ligne des soins dans la ville de Kisangani en République Démocratique du Congo : vers une typologie

**DOI:** 10.4102/phcfm.v13i1.2617

**Published:** 2021-09-06

**Authors:** Samuel I. Bosongo, Faustin C. Mukalenge, Albert M. Tambwe, Bart Criel

**Affiliations:** 1 Department of Public Health, Faculty of Medicine and Pharmacy, University of Kisangani, Kisangani, Democratic Republic of the Congo; 2 School of Public Health, Faculty of Medicine, University of Lubumbashi, Lubumbashi, Democratic Republic of the Congo; 3 Centre de Connaissances en Santé au Congo, Kinshasa, Democratic Republic of the Congo; 4 Department of Public Health, Institute of Tropical Medicine, Antwerp, Belgium

**Keywords:** physicians, first line healthcare facilities, health district system, typology, Democratic Republic of Congo

## Abstract

**Background:**

In the Democratic Republic of the Congo (DRC), for a number of years, there has been a spontaneous and growing phenomenon of physicians operating at the front line of the health system, while this role is traditionally devolved to nurse-practitioners. This phenomenon does not align with the current health policy.

**Aim:**

The aim of this paper is to develop and discuss the main types of frontline physicians in the city of Kisangani.

**Setting:**

We conducted a descriptive cross-sectional study in two urban districts in the city of Kisangani.

**Methods:**

The study population consisted of all first-line health facilities that employed at least one physician. The construction of a typology of first-line physicians consisted of three stages: identification and definition of relevant dimensions of analysis; grouping cases based on empirical data; and analysis of significant relationships and establishment of the typology itself.

**Results:**

An involvement of physicians in healthcare delivery at the first line was observed in 60% of all first line facilities in the two urban districts. Two main types of first-line physicians were identified: firstly, and by large the most prevalent one (96% of cases), the ‘hospital-like physician’, and secondly, the much less frequent type of the ‘supervision physician’.

**Conclusion:**

The involvement of physicians in first line healthcare is today a growing phenomenon in the DRC, especially in urban areas. The most dominant expression of this phenomenon is a transposition of the hospital-based physician model to the first line healthcare services, which thereby jeopardizing the specificity of first line healthcare.

## Introduction

Dans la majorité des pays d’Afrique sub-saharienne, les politiques de santé sont basées sur les Soins de Santé Primaires (SSP). Le District Sanitaire (DS), appelé Zone de Santé en République Démocratique du Congo (RDC), constitue le modèle dominant de leur opérationnalisation. Le DS est un système local de santé comprenant généralement deux lignes de soins en relation fonctionnelle: une première ligne constituée d’un réseau des structures qui offrent un paquet de soins de santé dynamique en réponse aux besoins ressentis par les populations locales et une deuxième ligne constituée d’au moins une structure offrant des soins de santé de référence, plus techniques et spécialisés.^1,2^

Suite à la pénurie en médecins durant la période postcoloniale, la majorité des pays africains, dont la RDC, a fait le choix de la délégation de certaines tâches médicales au personnel paramédical à la première ligne de soins.^3,4^ Loin d’être un pis-aller, cette délégation des tâches était une opportunité d’améliorer la couverture sanitaire, l’efficacité et l’efficience des soins grâce à la standardisation des tâches et à la supervision formative. Plusieurs études ont montré la pertinence de cette délégation des tâches non seulement dans les soins primaires en général,^5^ mais aussi dans la prise en charge des problèmes spécifiques de santé comme les maladies non transmissibles^6^ et le traitement antirétroviral.^7^

Actuellement, le contexte a sensiblement évolué en RDC. Le nombre de médecins produits est en nette augmentation suite notamment à l’augmentation du nombre de facultés de médecine. Ce nombre est passé de 3 en 1960 à 105 en 2017.^8^ En conséquence, alors que de 1962 à 2004 (43 ans), 6000 médecins étaient inscrits au tableau de l’Ordre National des Médecins de la RDC, plus de 19 000 médecins l’ont été de 2005 à 2017 (13 ans) étant donné que le dernier numéro attribué par l’Ordre National des Médecins dépassait déjà 25 000 en début 2017. Bien que le ratio soit de 1,06 médecin pour 10 000 habitants pour le pays,^9^ il existe une grande disparité caractérisée par la concentration de la majorité de ces médecins dans les centres urbains.

Paradoxalement, cette augmentation du nombre de médecins n’est pas accompagnée de l’augmentation du nombre (et/ou des capacités opérationnelles) des hôpitaux de référence sensés les utiliser conformément à la norme. Parallèlement, un secteur privé peu régulé se développe rapidement et domine l’offre à la première ligne de soins, particulièrement en milieu urbain. C’est ainsi qu’on assiste depuis quelques années au phénomène croissant de prestation à la première ligne de soins des médecins. Cette réalité ne trouve aujourd’hui pas sa place dans les documents de politique de santé du pays, créant ainsi un déphasage de la réalité par rapport à la norme.

Pourtant ailleurs (Europe, Amérique), le médecin a toujours été le principal responsable de la première ligne de soins. C’est pour valoriser cette pratique de médecine générale dans un contexte de spécialisation croissante que s’est développée la médecine de famille.^3^ En Afrique sub-saharienne, des modèles de prestation des médecins à la première ligne de soins, inspirés de la médecine de famille ont été rapportés dans certains pays notamment l’Afrique du Sud,^10,11^ le Mali, le Madagascar et le Bénin.^12,13^

Dans les pays du nord et certains pays du sud, la présence des médecins à la première ligne de soins est le résultat d’un processus organisé, caractérisé par une formation appropriée, une identité professionnelle valorisante et une régulation des prestations.^3^ En RDC cependant, elle constitue un phénomène spontané, non planifié, et probablement multiforme. Considéré par les uns comme un rétablissement de fait de l’ordre normal^14^ et par les autres comme un fait nouveau, ce phénomène demeure encore à ce jour largement sous documenté. L’objectif de cette étude était de développer et discuter de principaux types des médecins prestataires à la première ligne des soins existant dans la ville de Kisangani et formuler ainsi des recommandations pour la pratique et les politiques de santé.

## Méthodes

### Design de l’étude

Il s’agissait d’une étude transversale descriptive conduite au troisième trimestre de l’année 2018.

### Milieu d’étude

Notre étude s’est déroulée dans deux DS de la ville de Kisangani, en Province de la Tshopo au Nord-Est de la RDC. Il s’agissait des DS de Makiso-Kisangani et de Tshopo. Le DS de Makiso-Kisangani s’étend sur 801 km^2^ et comptait 344 789 habitants en 2018, soit une densité de 430 habitants/km^2^ tandis que le DS de la Tshopo a une superficie de 1553 km^2^ et sa population était estimée à 146 739 habitants en 2018, soit une densité de 94 habitants/km^2^. Cette faible densité du DS de Tshopo comparativement à celle de Makiso-Kisangani s’explique par le fait que sa partie péri-urbaine est plus étendue et moins peuplée.

Le DS de Makiso-Kisangani est subdivisée en 20 aires de santé, et celle de la Tshopo en 16 aires de santé. Les deux DS disposent d’un réseau des formations sanitaires de première ligne, des formations sanitaires de référence et des structures de coordination qui sont les Equipes Cadres de District (ECD).

### Population d’étude et stratégie d’échantillonnage

La population d’étude était constituée de toute formation sanitaire de première ligne qui bénéficiait des prestations d’au moins un médecin. Nous entendons par formation sanitaire de première ligne, tout établissement sanitaire moderne servant de premier contact de la population avec le système de santé et n’ayant pas à priori une vocation hospitalière ou spécialisée, qu’il soit public ou privé et quelle que soit sa dénomination (centre de santé, dispensaire, centre médical, etc.).

Les deux DS ont été sélectionnées de manière raisonnée pour des contraintes logistiques et de temps et parce qu’ayant presque les mêmes caractéristiques que les trois autres DS de la ville, leur situation serait représentative de l’ensemble de la ville. Au sein de chaque DS, nous avons inventorié toutes les formations sanitaires de première ligne. Leur identification a été faite au niveau des ECD, puis des aires de santé. Au niveau des ECD, les médecins chefs de district ont été contactés pour avoir le répertoire des aires de santé et des formations sanitaires de première ligne implantées dans chaque aire de santé. Au niveau des aires de santé, nous avions contacté les infirmiers titulaires pour se rassurer de l’existence ou non des formations sanitaires de première ligne répertoriées par les ECD et de l’existence d’autres formations sanitaires de première ligne non connues par les ECD. Les formations sanitaires de première ligne ainsi répertoriées ont été visitées par les enquêteurs pour s’enquérir des prestations ou non des médecins. Les responsables des formations sanitaires de première ligne bénéficiant des prestations des médecins ont été soumis à notre questionnaire après un consentement éclairé.

### Collecte et analyse des données

Les données ont été recueillies à l’aide d’un questionnaire préalablement testé. Ce questionnaire a été administré aux responsables des formations sanitaires de première ligne par cinq enquêteurs formés et supervisés sur terrain par le premier auteur. Pour construire la typologie des médecins prestataires à la première ligne de soins, nous avions recueilli les informations portant sur l’appartenance institutionnelle des formations sanitaires, le statut des médecins, le temps de ses prestations, la nature de ses prestations (curatives, préventives, promotionnelles, d’appui et de gestion), la tarification des soins et la collaboration avec l’ECD. Les données recueillies ont été saisies et traitées par le logiciel épi info 3.5.4 et l’analyse était essentiellement descriptive.

### Construction de la typologie

S’inspirant de Kluge,^15^ la construction de la typologie des médecins prestataires de première ligne dans notre étude s’est déroulée en trois étapes: (1) l’identification et la définition des dimensions pertinentes d’analyse, (2) le regroupement des cas à partir des données empiriques et (3) l’analyse des relations significatives et construction de types.

#### L’identification et la définition des dimensions pertinentes d’analyse

Six dimensions d’analyse ont été définies avant la collecte des données à partir de la littérature et de l’observation des pratiques sur terrain tel que détaillées ci-dessous:

L’**appartenance institutionnelle**: les formations sanitaires de première ligne ont été classées en trois catégories: publique, confessionnelle ou associative et privée indépendante (pour ne pas dire lucrative étant donné que les limites concernant le caractère lucratif entre les trois catégories ne sont plus évidentes dans le contexte d’autofinancement des formations sanitaires en RDC).^14^

Le **statut du médecin**: les médecins prestataires de première ligne ont été classés en quatre catégories selon l’observation du terrain : (1) médecin du secteur public ayant une affectation officielle dans une formation sanitaire de première ligne publique ou confessionnelle, (2) médecin du secteur public affecté officiellement dans un hôpital public ou dans une structure de l’administration sanitaire mais ayant un contrat formel (écrit et conforme au code du travail) ou informel (arrangement verbal) avec une formation sanitaire de première ligne privée indépendante, confessionnelle ou associative, (3) médecin du secteur privé ayant un contrat formel ou informel avec une formation sanitaire de première ligne privée indépendante, confessionnelle ou associative et (4) médecin délégué par une structure de coordination (ECD ou une structure confessionnelle ou associative).

Le **temps de prestation** a été classé en temps plein (chaque jour ouvrable pendant au moins 8 heures conformément à la législation congolaise) et en temps partiel (quelques jours ouvrables ou moins de 8 heures par jour) sur base des observations du terrain.

Le **paquet d’activités** constitué des soins curatifs, préventifs, promotionnels et des activités d’appui et de gestion (supervision, rapportage, monitoring…) conformément aux normes en vigueur en RDC. Nous nous sommes intéressés à l’implication effective des médecins dans ces différentes activités.

La **tarification des soins** et plus spécifiquement la différence ou non des tarifs des soins entre les médecins et les infirmiers dans la mesure où dans la totalité des formations sanitaires de première ligne, les médecins travaillent en équipe avec les infirmiers qui posent également des actes médicaux en leur absence, particulièrement dans les formations sanitaires de première ligne où les médecins prestent à temps partiel.

L’existence ou non d’une **collaboration avec l’ECD.** Cette collaboration se traduit par la reconnaissance officielle de la présence du médecin dans une formation sanitaire de première ligne par l’ECD, sa participation dans les supervisions et formations organisées par l’ECD dans la formation sanitaire comme les autres prestataires, ainsi que le rapportage des données issues de toutes ses activités.

#### Regroupement des cas

Sur base des données recueillies, les formations sanitaires de première ligne ont été groupées en fonction de leurs ressemblances majeures permettant ainsi d’identifier la première ébauche des types des médecins qui y prestent. Il convient de signaler qu’il ne s’agissait pas ici d’un shift des FOSA vers les médecins de première ligne. Nous nous sommes servis des FOSA pour analyser les prestations des médecins et tenter d’en tirer une typologie.

Les cas ainsi identifiés ont été comparés les uns aux autres afin de ressortir les ressemblances et les différences qui ont conduit à un deuxième regroupement, en veillant à ce que les éléments au sein de chaque groupe soient aussi similaires que possibles (homogénéité interne) et les différences entre les groupes aussi maximales que possibles (hétérogénéité externe).^15^

#### Analyse des relations significatives et construction de types

Le regroupement a permis la description des cas. Ensuite, un sens a été donné à chaque cas pour mieux le comprendre dans son contexte.^15^

### Considérations éthiques

Le protocole de cette étude a été approuvé par le comité d’éthique médicale de l’Université de Lubumbashi le 14/06/2018 (N° Approbation: UNILU/CEM/092/2018). Un formulaire écrit de consentement éclairé garantissant l’anonymat et la libre participation a été proposé aux responsables de toutes les structures identifiées avant à l’enquête.

## Résultats

### Ampleur et caractéristiques des formations sanitaires de première ligne «médicalisées» à Kisangani

Au total 98 formations sanitaires de première ligne ont été identifiées dans les 36 aires de santé des deux DS d’étude. Parmi elles, 53 (soit 60%) bénéficiaient des prestations d’au moins un médecin et 51 (soit 92.3%) ont consenti à l’étude. Ces formations sanitaires de première ligne appartenaient en majorité au secteur privé indépendant (78%) dont les propriétaires étaient notamment les infirmiers (45%) et les médecins (38%). Si la majorité de ces formations sanitaires de première ligne (98%) étaient connues au niveau des DS, plus de trois quarts des médecins qui y prestaient (79%) n’étaient pas reconnus comme prestataires à la première ligne de soins par les ECD et leurs prestations étaient dissimulées et non rapportées.

### Caractéristiques générales des prestations des médecins à la première ligne

Le Tableau 1 montre qu’indépendamment de leur appartenance institutionnelle, la majorité des formations sanitaires de première ligne bénéficiaient des prestations des médecins à temps partiel. Ces prestations étaient principalement curatives (dominées par des actes hospitaliers comme l’hospitalisation, les interventions chirurgicales majeures, la transfusion sanguine, etc.) et à moindre mesure préventives, promotionnelles ou d’appui et de gestion. La tarification par acte était dominante et les tarifs des médecins étaient généralement supérieurs à ceux des infirmiers.

**TABLEAU 1:** Caractéristiques des prestations des médecins à la première ligne des soins.

Dimensions d’analyse	Secteur public (*n* = 5)		Secteur confessionnel et associatif (*n* = 6)		Secteur privé indépendant (*n* = 40)		Total (*n* = 51)	
	*n*	%	*n*	%	*n*	%	*n*	%
**Statut du médecin**								
Médecin de l’État affecté officiellement	5	100	0	0	0	0	**5**	**10**
Médecin de l’État ayant un contrat (formel ou informel) avec la structure	0	0	3	50	22	55	**25**	**49**
Médecin privé ayant un contrat (formel ou informel) avec la structure	0	0	1	17	18	45	**19**	**37**
Médecin délégué par l’ECD ou la structure de coordination médicale de l’église ou association	0	0	2	33	0	0	**2**	**4**
**Temps de prestation**								
Partiel	3	60	5	83	22	55	**30**	**59**
Plein	2	40	1	17	18	45	**21**	**41**
**Tarification des soins**								
**Mode de tarification dans les formations sanitaires de première ligne**								
Forfaitaire	3	60	3	50	9	23	**15**	**29**
Par acte	2	40	3	50	31	77	**36**	**71**
**Différence de tarif entre médecins et infirmiers**								
Supérieure à celui des infirmiers	1	20	3	50	27	67	**31**	**61**
Egale à celui des infirmiers	4	80	3	50	13	33	**20**	**39**
**Collaboration avec l’ECD**								
Oui	5	100	4	67	7	18	**16**	**31**
Non	0	0	2	33	33	72	**35**	**79**
**Paquet d’activités**								
**Activités curatives**								
Consultations curatives ambulatoires	5	100	6	100	40	100	**51**	**100**
Hospitalisation	4	80	4	67	34	85	**42**	**82**
Accouchements dystociques	4	80	4	67	31	78	**39**	**77**
Chirurgie majeure	3	60	6	100	32	80	**41**	**80**
Transfusion sanguine	3	60	4	67	27	68	**34**	**67**
**Activités préventives et promotionnelles**								
Consultations prénatales	4	80	3	50	24	60	**31**	**61**
Consultations post-natales	3	60	3	50	20	50	**26**	**51**
Planification familiale	3	60	0	0	13	32	**16**	**31**
Consultations préscolaires/vaccinations	3	60	2	33	12	30	**17**	**33**
Promotion de la santé	3	60	3	50	13	33	**19**	**37**
**Activités d’appui et de gestion**								
Micro planification	0	0	1	17	7	18	**8**	**16**
Rapportage des données	0	0	1	17	12	30	**13**	**26**
Monitorage des activités	0	0	1	17	12	30	**13**	**26**
Supervision des autres prestataires	0	0	2	33	20	50	**22**	**43**
Visites à domicile	0	0	1	17	7	18	**8**	**16**
Supervision des relais communautaires	0	0	0	0	0	0	**0**	**0**
Implication dans le comité de santé	0	0	1	17	0	0	**1**	**2**

### Typologie des médecins prestataires à la première ligne

Cinq cas de figure des médecins prestataires de première ligne ont été identifiés après regroupement des formations sanitaires sur base de leurs ressemblances à partir des données empiriques (Tableau 2). Il s’agissait des cas suivants :

**TABLEAU 2:** Cas de figure des médecins à la première ligne à Kisangani.

Dimensions	Secteur Public Cas 1 (*n* = 5)	Secteur privé indépendant (*n* = 40)		Secteur privé confessionnel et associatif (*n* = 6)	
		Cas 2 (*n* = 22)	Cas 3 (*n* = 18)	Cas 4 (*n* = 4)	Cas 5 (*n* = 2)
Statut du médecin	Médecin affecté officiellement	Médecin du secteur public ayant un contrat avec la structure	Médecin du secteur privé ayant un contrat avec la structure	Médecin du secteur public ou privé ayant un contrat avec la structure	Médecin délégué par la structure de coordination de l’église ou de l’association
Temps de prestation	Temps plein ou temps partiel	Temps partiel	Temps partiel ou temps plein	Temps partiel	Temps partiel
Paquet d’activités	Principalement curative hospitalière	Principalement curative hospitalière	Principalement curative hospitalière	Principalement curative hospitalière	Principalement Appui (supervision avec démonstration)
	Parfois préventif et promotionnel	Parfois préventif et promotionnel	Parfois préventif et promotionnel	Parfois préventif et promotionnel	
		A moindre mesure appui et gestion	A moindre mesure appui et gestion	A moindre mesure appui et gestion	
Tarification des soins	Généralement supérieure aux infirmiers	Généralement supérieure aux infirmiers	Généralement supérieure aux infirmiers	Généralement supérieurs à l’infirmier	Mêmes que l’infirmier
			Parfois même que les infirmiers	Parfois même que les infirmiers	
Collaboration avec l’ECZ	Oui	Non	Généralement Non Parfois Oui	Non	Oui

Le **médecin prestataire public** (cas 1) : c’est un médecin affecté officiellement dans une formation sanitaire de première ligne étatique ou paraétatique. Il preste généralement à temps plein et ses prestations sont principalement curatives et dominées par des actes hospitaliers (tels que chirurgie majeure, hospitalisation, transfusion sanguine, etc.) avec moins d’intérêt aux activités préventives et promotionnelles. La tarification des soins ne diffère généralement pas de celle appliquée par les infirmiers. Il est reconnu par l’ECD avec laquelle il collabore.

Le **médecin prestataire avec double emploi public-privé** (cas 2 et 4) : c’est un médecin employé par l’Etat dans un hôpital public ou dans une structure de l’administration sanitaire (notamment la division et l’inspection provinciales de la santé) qui preste généralement à temps partiel dans une structure privée. Son paquet d’activités est essentiellement curatif et dominé par des actes hospitaliers (tels que chirurgie majeure, hospitalisation, transfusions sanguines etc.) et dans une moindre mesure préventif et promotionnel. Ses tarifs des soins sont généralement supérieurs à ceux des infirmiers. Il n’est pas officiellement reconnu par l’ECD et par conséquent n’entretient aucune relation avec elle.

Le **médecin prestataire privé** (cas 3) : il s’agit d’un médecin privé ou en attente d’affection dans une structure étatique qui preste dans une formation sanitaire privée à temps plein ou à temps partiel et y applique un paquet de soins essentiellement curatif et parfois préventif et promotionnel en fonction de son temps de prestation. La tarification est généralement supérieure à celle appliquée par les infirmiers et parfois la même. Il n’est pas officiellement reconnu par l’ECD et par conséquent n’entretient aucune relation avec elle.

Le **médecin délégué** (cas 5) : il s’agit d’un médecin délégué par une structure de coordination médicale de l’église ou de l’association qui preste à temps partiel dans une FOSA confessionnelle ou associative et apporte en plus de ces prestations, un appui technique à l’infirmier dans le cadre de la délégation des tâches. Cet appui se fait soit sur demande de l’infirmier ou soit à sa propre initiative en fonction des déficiences constatées. Ses prestations se limitent au paquet minimum d’activités reconnu à la première ligne et n’entrainent aucune modification de la tarification des soins. Il est officiellement reconnu par l’ECD avec laquelle il entretient une étroite collaboration.

**FIGURE 1:**
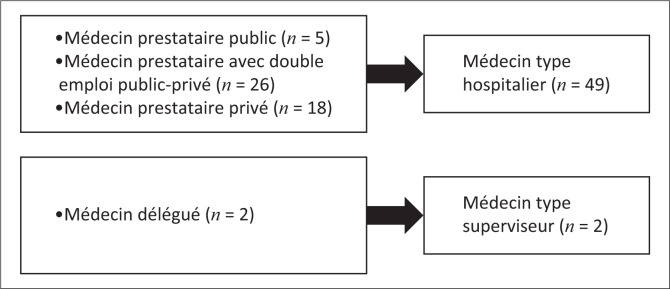
Types des médecins prestataires en première ligne de soins à Kisangani.

L’analyse des ressemblances et différences de ces cinq cas a conduit à leur regroupement en deux principaux types (Figure 1) et l’analyse de leur signification en fonction du contexte congolais nous a amené à les caractériser comme suit:

Le premier est le **«médecin type hospitalier»** dont les prestations sont essentiellement curatives de type hospitalier allant au-delà du paquet minimum d’activités reconnu à la première ligne de soins par les normes du pays. Ses tarifs sont généralement supérieurs à ceux appliqués par les infirmiers. Il ne collabore pas avec l’ECD qui ne le reconnait pas sauf s’il preste dans le secteur public (cas 1). C’est le modèle dominant (observé dans 49 formations sanitaires de première ligne sur 51, soit 96%) dans tous les sous-secteurs du système de santé (public, privé confessionnel ou associatif et indépendant). Il comprend le médecin prestataire public (cas 1), le médecin prestataire avec double emploi public-privé (cas 2 et 4) et le médecin prestataire privé (cas 3).

Le second est le **«médecin type superviseur»** dont les prestations sont conformes au paquet minimum d’activités en vigueur et visent à renforcer les compétences des infirmiers dans le cadre de la délégation des tâches sans influer sur la tarification. Il est reconnu par l’ECD et collabore avec elle. Ce type est minoritaire à Kisangani (dans 2 formations sanitaires de première ligne sur 51, soit 4%) et n’a été observé que dans le sous-secteur confessionnel ou associatif. Il est constitué du médecin délégué d’une structure de coordination médicale de l’église ou de l’association (cas 5).

## Discussion

Notre étude qui visait à établir une typologie des médecins prestataires de première ligne de soins a permis d’en identifier deux types, à savoir: le ‘médecin type hospitalier’ qui est dominant et le ‘médecin type superviseur’ qui est rare.

### Ampleur et caractéristiques des formations sanitaires de première ligne «médicalisées» à Kisangani

Notre étude a montré que la présence du médecins prestataires de première ligne était une réalité à Kisangani où 60% des formations sanitaires de première ligne était ‘médicalisées’. Ce résultat corrobore celui de Chenge et al.^14^ à Lubumbashi où 2/3 des formations sanitaires de première ligne comptaient parmi leurs personnels au moins un médecin.

Il est ressorti de notre étude que trois quarts des formations sanitaires de première ligne médicalisées se recrutaient dans le secteur privé indépendant. Cette observation a été aussi faite par Chenge et al.^14^ à Lubumbashi en RDC et Cadot et al.^16^ à Ouagadougou au Burkina-Faso. Cette similitude des résultats se justifie par la prédominance de ce secteur dans les villes pour des raisons lucratives.

### Caractéristiques générales des prestations des médecins à la première ligne

Nous avons observé que le double-emploi public-privé et privé-privé caractérisait les médecins prestataires de première ligne. Nous pensons qu’il s’agit d’une part de stratégie de survie individuelle face à la faible rémunération; et d’autre part de stratégie de maintien de compétence face à la pléthore des médecins dans les hôpitaux publics sous-utilisés qui réduit le temps et le volume de prestation. Ce double-emploi justifierait la prédominance des prestations à temps partiel.

Nos résultats ont montré également que la présence du médecin prestataire de première ligne était associée à l’augmentation des tarifs des soins. Nous pensons que cette augmentation serait susceptible d’impacter négativement sur l’accès et l’équité des soins. Aussi, il a été observé que la majorité des médecins était rémunérée à l’acte. Cette situation pourrait constituer une source de marchandisation des soins et justifierait en partie leur préférence aux actes hospitaliers réputés rentables comme les interventions chirurgicales.

Notre étude a montré que trois quarts des médecins prestataires à la première ligne de soins n’étaient pas reconnus comme tel par les ECD. En réalité, il s’agit d’un secret de polichinelle qui illustre bien la notion de norme pratique’ définie par Olivier de Sardan.^17^

### Typologie des médecins prestataires à la première ligne

Deux principaux types de médecins prestataires de première ligne ont été identifiés au cours de notre étude, à savoir le ‘médecin type hospitalier’ qui est dominant et le ‘médecin type superviseur’ qui est rare.

Le médecin type superviseur correspond à un des six rôles assignés aux médecins de famille en Afrique du Sud, à savoir ‘clinical outreach and support to primary care clinics and health centres’.^10^ Théoriquement, ce modèle aurait un potentiel de renforcement du DS dans la mesure où il favorise le renforcement des capacités des infirmiers à la première ligne de soins par les supervisions régulières et préserve la complémentarité entre les deux échelons des soins. Ce type a été prévu dans la législation sanitaire congolaise qui exige pour chaque formation sanitaire privée, un médecin superviseur chargé de veiller à la qualité des prestations du personnel paraclinique. Il est à encourager dans le contexte de la délégation des taches médicales.^5^ Cependant, la rareté observée sur terrain se justifierait entre autres par l’impréparation des médecins à jouer ce rôle. D’où la nécessité de l’adaptation de la formation telle que proposée par Eyal et al.^18^

Le médecin type hospitalier par contre, aurait un très faible potentiel de renforcement du DS. Il contribue non seulement à la dénaturation de la première ligne de soins en transformant ses formations sanitaires en ‘petits hôpitaux’ qui perdent ainsi leur spécificité^4^ mais aussi à la désintégration du DS en créant une compétition entre ses deux échelons. Le faible taux de référence (1.2%), la faible proportion des malades référés en hospitalisation (11.4%) et la faible proportion de césarienne (2.3%) rapportés en 2018 dans les 5 DS de la ville de Kisangani illustre bien cette compétition (données tirées du rapport de la revue annuelle 2018 de la division provinciale de la santé de Tshopo). En outre, la réalisation des actes hospitaliers (interventions chirurgicales notamment) dans les formations sanitaires de première ligne dont la grande majorité ne dispose pas d’infrastructure adéquate, entame de façon considérable la qualité de ces actes et la sécurité des patients.

Nous pensons que cette dérive hospitalière à la première ligne de soins est la résultante d’une conjonction de plusieurs facteurs. Il s’agit notamment de (1) l’hospitalocentrisme hérité du système colonial, la médecine générale de premier échelon n’ayant pas été importée dans les colonies^19^; (2) l’inadéquation entre la formation hospitalière des médecins et les spécificités de la première ligne de soins^1,3,19^; (3) la survie institutionnelle face au désengagement de l’Etat dans l’allocation des ressources contraignant les formations sanitaires à fonctionner dans une logique commerciale^20^ que publique^21^; (4) l’insuffisance de régulation du système de santé avec comme corollaire les ouvertures incontrôlées des structures privées des soins ne répondant pas aux normes et (5) le construit social de l’image du médecin, considéré par la majorité de la population congolaise comme ‘celui qui opère’.

### Le médecin à la première ligne: vers un modèle optimal?

En dépit de la dérive hospitalière démontrée ci-haut, nous faisons l’hypothèse qu’il est possible d’évoluer vers un modèle plus optimal, inspiré de la médecine de famille. Pour y arriver, certains préalables devraient être réunis, notamment (1) l’adaptation de la formation des médecins aux spécificités de la première ligne de soins^3,19^; (2) le renforcement de la régulation du système de santé; (3) la révision du paquet minimum d’activités de manière à élargir la gamme de problèmes de santé à prendre en charge à la première ligne de soins; (4) la clarification des rôles entre médecins et infirmiers et (5) la débureaucratisation des relations entre les ECD et les formations sanitaires de première ligne pour privilégier l’innovation et le professionnalisme.^3^

### Limites de l’étude

Ce travail comporte certaines limites d’ordre méthodologique qu’il convient de reconnaître. La première est relative à la sélection des formations sanitaires. L’identification des formations sanitaires de première ligne à travers les structures administratives (notamment les ECD) pourrait constituer des sources possibles de biais de sélection dans la mesure où il était possible de passer à côté des structures nouvellement créées et/ou non en règle avec l’administration sanitaire. Cependant, ce biais a été minimisé par la technique de boule de neige qui consistait à s’enquérir systématiquement auprès du responsable et/ou d’un prestataire de chaque formation sanitaire visitée de l’existence ou non d’une autre formation sanitaire proche. La seconde limite concerne la construction de la typologie des médecins prestataires de première ligne. Nous reconnaissons que la liste des dimensions d’analyse sur base desquelles était basée notre typologie n’est pas exhaustive et que certaines dimensions étaient relativement peu discriminatoires. Il existerait plusieurs autres dimensions qui pourraient entrer en jeu mais nous nous sommes limités à celles jugées pertinentes pour notre milieu d’étude.

## Conclusion

La prestation des médecins à la première ligne de soins est aujourd’hui une réalité croissante en milieu urbain en RDC. Son expression la plus dominante consiste en une tentative de transposition du modèle hospitalier à la première ligne de soins qui perd ainsi sa spécificité et se retrouve dénaturée. Cette situation est la résultante d’une combinaison de facteurs, à savoir l’hospitalocentrisme hérité de la colonisation, l’inadéquation de la formation actuelle des médecins à l’exercice d’une médecine de première ligne holistique, une très forte logique de survie institutionnelle et individuelle avec des dérives vers une franche commercialisation des soins au détriment de l’accès et de la qualité, l’insuffisance de régulation et le construit social de l’image du médecin. Il y a dès lors nécessité d’étudier davantage l’influence que ce phénomène aurait sur le système de santé de district en tant que système intégré, ainsi que la plus-value éventuelle des prestations des médecins à la première ligne de soins en termes de qualité des soins. En outre, une étude similaire en milieu rural serait intéressante pour avoir une image plus large et complète qui peut influencer les changements des politiques au niveau national.
